# Behavioral and neural underpinnings of empathic characteristics in a Humanitude-care expert

**DOI:** 10.3389/fmed.2023.1059203

**Published:** 2023-05-25

**Authors:** Wataru Sato, Atsushi Nakazawa, Sakiko Yoshikawa, Takanori Kochiyama, Miwako Honda, Yves Gineste

**Affiliations:** ^1^Psychological Process Research Team, Guardian Robot Project, RIKEN, Soraku-gun, Japan; ^2^Graduate School of Interdisciplinary Science and Engineering in Health Systems, Okayama University, Okayama, Japan; ^3^Kyoto University of the Arts, Kyoto, Japan; ^4^Brain Activity Imaging Center, ATR-Promotions Inc., Soraku-gun, Japan; ^5^Department of Geriatric Medicine, National Hospital Organization Tokyo Medical Center, Tokyo, Japan; ^6^IGM-France, Saint-Laurent-de-la-Salanque, France

**Keywords:** empathy, expert, facial mimicry, fMRI, Humanitude care, mirror neuron system

## Abstract

**Background:**

Humanitude approaches have shown positive effects in elderly care. However, the behavioral and neural underpinnings of empathic characteristics in Humanitude-care experts remain unknown.

**Methods:**

We investigated the empathic characteristics of a Humanitude-care expert (YG) and those of age-, sex-, and race-matched controls (*n* = 13). In a behavioral study, we measured subjective valence and arousal ratings and facial electromyography (EMG) of the corrugator supercilii and zygomatic major muscles while participants passively observed dynamic facial expressions associated with anger and happiness and their randomized mosaic patterns. In a functional magnetic resonance imaging (MRI) study, we measured brain activity while participants passively observed the same dynamic facial expressions and mosaics. In a structural MRI study, we acquired structural MRI data and analyzed gray matter volume.

**Results:**

Our behavioral data showed that YG experienced higher subjective arousal and showed stronger facial EMG activity congruent with stimulus facial expressions compared with controls. The functional MRI data demonstrated that YG showed stronger activity in the ventral premotor cortex (PMv; covering the precentral gyrus and inferior frontal gyrus) and posterior middle temporal gyrus in the right hemisphere in response to dynamic facial expressions versus dynamic mosaics compared with controls. The structural MRI data revealed higher regional gray matter volume in the right PMv in YG than in controls.

**Conclusion:**

These results suggest that Humanitude-care experts have behavioral and neural characteristics associated with empathic social interactions.

## 1. Introduction

Given the increasing numbers of people with dementia worldwide (1), Humanitude care has attracted interest. Humanitude care was developed by Gineste and Marescotti in 1979 as relationship-centered care for people with dementia (2). This methodology facilitates gentle, positive interaction with patients with dementia (3) using more than 150 social skills, such as looking at them face-to-face (4, 5). Several studies have shown that Humanitude care effectively reduces behavioral and psychological symptoms of dementia in patients [e.g., (6, 7); for a review, see (8)]. Furthermore, studies showed that the experiences of Humanitude caregivers enhance empathy [i.e., the ability to respond emotionally to and understand others’ emotions (9)] (10, 11). These data suggest that Humanitude care has positive effects on both patients and caregivers, which promotes the necessity of research on this technique.

However, the characteristics of Humanitude-care experts remain unknown. Such information would deepen understanding of Humanitude care and facilitate its application. We hypothesized that Humanitude-care experts could have behavioral and neural characteristics associated with empathy, based on findings that Humanitude care experiences increase empathy in caregivers (10, 11). Several behavioral studies have shown that motor synchrony, such as facial mimicry, underlies empathic processing, including both sharing and recognizing the emotional states of others [e.g., (12); for reviews, see (13–15)]. Neuroscientific studies have suggested that the mirror neuron system (MNS) may underlie such empathic interaction via motor synchronization [for reviews, see (16–19)]. Among the core regions of the MNS, including the ventral premotor cortex (PMv; including the precentral and inferior frontal gyri), inferior parietal lobule, and superior temporal sulcus (STS) region (including the posterior middle and superior temporal gyri) (20), dynamic face-to-face interaction specifically activates the PMv and STS region in the right hemisphere (21–24). We hypothesized that Humanitude-care experts, compared with non-experts, would show enhanced facial mimicry of other individuals’ facial expressions, and have increased functional and structural neural substrates in the MNS.

To test this hypothesis, we conducted a case study of one Humanitude-care expert, YG, who was a Caucasian male trained in Humanitude care for more than 38 years. We conducted a series of behavioral, functional magnetic resonance imaging (MRI), and structural MRI studies with YG and age-, sex-, and race-matched controls. In the behavioral study, to test subjective empathic responses and facial mimicry, we measured subjective emotional ratings and facial electromyography (EMG) while participants passively observed dynamic facial expressions associated with anger and happiness and their randomized mosaic patterns. To test MNS activity, we conducted a functional MRI study measuring brain activity while participants passively observed the same dynamic facial expressions and mosaics. We further conducted a structural MRI study to analyze the structural characteristics of the MNS. We used the dynamic facial expression stimuli of Japanese models that were previously shown to elicit facial mimicry and MNS activity (23, 25, 26). In addition, as previous behavioral studies reported that the effects of trait empathy were more evident on attitudes toward outgroups than on those to ingroups (27, 28), we expected that the faces of people who differed racially from the participants would reveal the empathic characteristics of YG.

## 2. Materials and methods

### 2.1. Participants

YG was a 63-year-old Caucasian male who was one of the founders of Humanitude care. YG was an exceptional expert (29) of Humanitude care who worked at the master level in the objective criteria of teaching and setting standards (30). He has been involved in Humanitude care for more than 38 years. He previously worked as a caregiver and an educator for caregivers, caring for more than 30,000 people with dementia (about 40 per week). He used the techniques of Humanitude care, developed during his own caring experience, together with that of a colleague. Humanitude care features a structured sequence of caring procedures, based on the four pillars of gaze, speech, touch, and verticality, each of which includes many operationalized skills (4). When the experiment was conducted, he worked principally as an educator, delivering lectures on Humanitude care to about 500 institutes annually. The first language of YG was not Japanese.

The control group included 13 Caucasian male adults (mean ± *SD* age, 54.5 ± 9.4 years); the participants were matched with YG for age [Crawford and Howell’s (31) modified *t*-test, *t* = 0.88, *p*(two-tailed) = 0.394, *z*_*cc*_ = 0.92], sex, and race. Control participants were recruited from a local human resource company (Chuo Sato, Amagasaki, Japan) through advertisements offering temporary jobs to foreigners in the West Japan region. The inclusion criteria were Caucasian race, male sex, age between 40 and 69 years, and a willingness to participate in behavioral and MRI studies at Kyoto University, Japan. The exclusion criteria included any contraindication to MRI (e.g., a pacemaker). The first language of all controls was other than Japanese. The sample size was determined by reference to the result of a power analysis using Crawford and Howell’s modified *t*-test (31) that detecting a deviation > 3 *SD* in the case with a power of 0.84 requires 10 controls (32). Also, we heuristically (33) referred to a previous functional MRI study that compared a sports expert with a control group of ordinary sports players (*n* = 6) (34).

All participants had normal or corrected-to-normal visual acuity. Following an explanation of the experimental procedure, all participants gave informed consent. This study was approved by the Ethics Committee of the Unit for Advanced Studies of the Human Mind, Kyoto University, Japan. All experiments complied with institutional ethical provisions and the Declaration of Helsinki.

### 2.2. Stimuli

**FIGURE 1:**
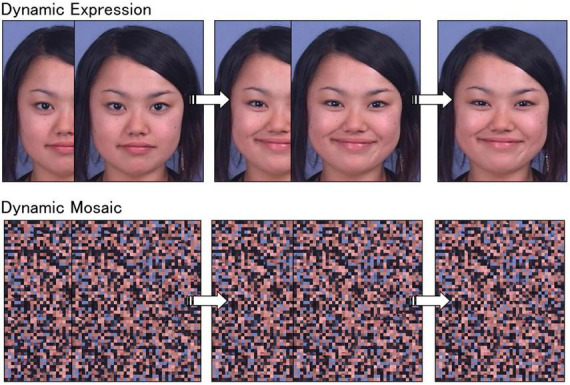
Illustrations of dynamic facial expression and dynamic mosaic stimuli.

For the behavioral and functional MRI studies, video clips of angry and happy facial expressions by four Japanese women and four Japanese men were used as dynamic facial expression stimuli. These stimuli were selected from our video database of facial expressions of emotion, which included expressions by 65 Japanese models (35). The stimulus models looked straight ahead and were unfamiliar to the participants. These specific stimulus expressions were selected because they represented theoretically appropriate facial expressions, as confirmed by coding analyses performed by a trained coder using the Facial Action Coding System (36) and the Facial Action Coding System Affect Interpretation Dictionary (37). The speeds of dynamic changes in these expressions were within the natural range of the observers (38), and the stimuli were validated in several previous behavioral and functional MRI studies. Specifically, the stimuli elicited appropriate subjective emotional responses (39) and spontaneous facial mimicry (25, 26) and activated the MNS, including the right PMv and the bilateral STS regions (23). The dynamic expression stimuli comprised 38 frames ranging from neutral to emotional expressions. Each frame was presented for 40 ms and each clip for 1,520 ms. The stimuli subtended a visual angle of approximately 15° vertical × 12° horizontal. An example of the stimulus sequence is shown in Figure 1, which contains data from a model who provided consent for the use of her image in scientific publications.

To create dynamic mosaic image stimuli, all dynamic facial expression frames were divided into 50 vertical × 40 horizontal squares and rearranged using a fixed randomization algorithm (Figure 1). This procedure made each image unrecognizable as a face. A set of these 38 frames was serially presented as a moving clip, corresponding to the original dynamic face images, at the same speed as that for the dynamic expression stimuli. As a result, dynamic mosaic stimuli were presented with smooth motion comparable to natural dynamic facial expressions, even though they were unrecognizable as faces.

### 2.3. Presentation apparatus

For the behavioral and functional MRI studies, the experiments were controlled using the Presentation software (Neurobehavioral Systems, Albany, CA, USA). In the behavioral study, the stimuli were presented on a 19-inch cathode ray tube monitor (HM903D-A, Iiyama, Tokyo, Japan). In the functional MRI study, the stimuli were projected from a liquid crystal projector (DLA-HD10K; Japan Victor Company, Yokohama, Japan) to a mirror positioned in front of the participants.

### 2.4. Procedure

The studies were conducted individually. The behavioral study was conducted first, followed by the functional and structural MRI studies.

#### 2.4.1. Behavioral study

The behavioral study was conducted using procedures described previously (26), with some modifications. An electrically shielded soundproof room was used for the experiments. At the beginning of the experiments, participants were told that the experiment involved recording electric activity from the skin to conceal the real purpose of our muscle activity tests. After electrode placement, the participants were told to view the stimuli and then evaluate them. EMG recordings were conducted while the participants passively viewed the stimuli. A total of 32 trials were performed, consisting of eight trials each of angry faces, happy faces, angry mosaics, and happy mosaics. The stimuli were presented in random order.

In each trial, a fixation point (a small gray cross on a white background) was presented at the center of the screen for 1,520 ms, and then the stimulus was presented for 1,520 ms. Next, the screen was filled with a solid gray field as an inter-trial interval, with length varying randomly between 6,000 and 9,000 ms.

After the EMG recordings, the stimuli were presented again, and the participants were asked to respond to the question, “How did you feel emotionally when you viewed the expression?” using an affect grid (40) that graphically assessed the two dimensions of valence and arousal on nine-point scales. Valence and arousal ranged from −4 (negative) to + 4 (positive) and from −4 (low arousal) to + 4 (high arousal), respectively. The general interpretation is that valence represents the qualitative component and arousal reflects the energy of either positive or negative emotions (41). The stimuli were presented in random order.

#### 2.4.2. Functional MRI study

The functional MRI study was conducted as described previously (23), with modifications. Each participant completed a single functional MRI scan consisting of 20 epochs of 20 s each, separated by 20 rest periods (a blank screen) of 10 s each. Each of the four stimulus conditions was presented in different epochs within each run, and the order of epochs was pseudorandomized. The order of stimuli within each epoch was randomized. Each epoch consisted of eight trials, and a total of 160 trials were performed by each participant. Stimulus trials were replaced by target trials in eight trials (two trials in each of the angry dynamic facial expression, happy dynamic facial expression, angry dynamic mosaic, and happy dynamic mosaic conditions).

In each stimulus trial, a fixation point (a small gray cross on a white background) was presented in the center of the screen for 980 ms, followed by the stimulus for 1,520 ms. In each target trial, a red cross (approximately 1.2° × 1.2°) was presented instead of the stimulus. Participants were asked to press a button using their right index fingers as quickly as possible when a red cross appeared and to gaze at the fixation point in each trial; they received no other information (e.g., stimulus type). These dummy tasks were conducted to ensure that the participants attended to the stimuli but did not engage in either controlled processing of the stimuli or stimulus-related motor responses.

After functional and structural image acquisition, the participants were interviewed to determine whether they had been aware that their muscle activity and related brain activity/structure had been tested. This process ensured that all participants, including YG, had been unaware of the research purpose. Debriefing was conducted, and then participant permission to use their data for analysis was requested and granted for all participants.

### 2.5. Measurement

#### 2.5.1. EMG

EMG was used to monitor the muscles of the corrugator supercilii (related to brow-lowering actions, prototypical of angry facial expressions) and the zygomatic major (related to lip-corner-pulling actions, prototypical of happy facial expressions). These muscles were selected as indices of facial mimicry because several previous studies employing facial EMG indicated that observation of angry and happy facial expressions induced corrugator supercilii and zygomatic major muscle activities, respectively [e.g., (42)]. The Ag/AgCl electrodes were placed according to established guidelines (43, 44). A ground electrode was placed on the forehead. The data were amplified, filtered online (band pass: 20–400 Hz), and sampled at 1,000 Hz using an EMG-025 amplifier (Harada Electronic Industry, Sapporo, Japan) and the PowerLab 16/35 data acquisition system and LabChart Pro v8.0 software (ADInstruments, Dunedin, New Zealand). A low-cut filter at 20 Hz was applied because it has been reported to remove motion artifacts from facial EMG (45). All participants were video monitored using an unobtrusive digital web camera (HD1080P; Logicool, Tokyo, Japan).

#### 2.5.2. Functional MRI

Functional and structural image scanning was performed on a 3T scanning system (MAGNETOM Verio; Siemens, Malvern, PA, USA) using a 32-channel head coil. Elastic pads were used to stabilize the participants’ head position. The functional images consisted of 40 consecutive slices parallel to the anterior–posterior commissure plane, covering the whole brain. A T2*-weighted gradient-echo echo-planar imaging sequence was used with the following parameters: repetition time (TR) = 2,500 ms; echo time (TE) = 30 ms; flip angle = 90°; matrix size = 64 × 64; and voxel size = 3 × 3 × 4 mm. The slices were in ascending order.

#### 2.5.3. Structural MRI

Following the acquisition of functional images, a T1-weighted, high-resolution structural image was acquired using a magnetization-prepared rapid-acquisition gradient-echo sequence (TR = 2,250 ms; TE = 3.06 ms; inversion time = 1,000 ms; flip angle = 9°; field of view = 256 × 256 mm; voxel size = 1 × 1 × 1 mm).

### 2.6. Data analyses

#### 2.6.1. Subjective ratings

The valence and arousal ratings were analyzed separately. The ratings of dynamic angry expression, dynamic happy expression, and dynamic mosaic stimuli were averaged separately for each participant. Composite scores were calculated by averaging valence ratings for angry expressions × −1 and valence ratings for happy expressions to represent valence and averaging arousal ratings for angry and happy expressions to represent arousal. The composite scores were analyzed in terms of the difference between YG and controls using Crawford and Howell’s modified *t*-test (31, 46) (one-tailed) implemented using the Singlims_ES function (47). This test is a modified version of the two-sample *t*-test in which the target single case is treated as a sample of *n* = 1 and does not contribute to estimating the within-group variance (31). A result was considered statistically significant at *p* < 0.05.

#### 2.6.2. EMG

EMG data were analyzed using the Psychophysiological Analysis Software 3.3 (Computational Neuroscience Laboratory of the Salk Institute, La Jolla, CA, USA) implemented in MATLAB 2020a (MathWorks, Natick, MA, USA). The data were sampled for 3,500 ms in each trial, including pre-stimulus baseline data for 1,000 ms (during observation of the fixation point) and the data for 2,500 ms after stimulus onset. The time window of the post-stimulus period was the same as that of a previous study that detected facial EMG activity in response to dynamic facial expressions (26). For each trial, the differences in the mean absolute amplitudes between the pre- and post-stimulus periods were calculated as the EMG data.

As in the subjective rating analysis, the mean EMG activity was calculated for each condition of each participant, excluding data beyond the total mean > 3 *SD* or < −3 *SD* as artifacts. Then, as a measure of facial mimicry, corrugator supercilii EMG activity in response to angry expressions and zygomatic major EMG activity in response to happy expressions were analyzed using Crawford and Howell’s (31) modified *t*-test (one-tailed).

#### 2.6.3. Functional MRI

Functional and structural MRI analyses were performed using the statistical parametric mapping package SPM12,^1^ implemented in MATLAB R2020a (MathWorks, Natick, MA, USA).

As a pre-processing step, functional images of each run were first realigned with the first scan as a reference to correct for head motion. The realignment parameters revealed only a small (< 2 mm) motion correction. All functional images were corrected for slice timing, and then the functional images were coregistered to the anatomical image. Next, all anatomical and functional images were normalized to Montreal Neurological Institute (MNI) space using the anatomical image-based unified segmentation–spatial normalization approach (48). Finally, the spatially normalized functional images were resampled to a voxel size of 2 × 2 × 2 mm and smoothed with an isotropic Gaussian kernel of 8-mm full-width at half-maximum (FWHM) to compensate for anatomical variability among participants.

Random-effects analyses were performed to identify significantly activated voxels at the population level (49). First, a single-subject analysis was performed (50). The task-related regressor for each stimulus condition and target condition was modeled by the boxcar and delta functions, respectively, convolving it with a canonical hemodynamic response function for each presentation condition in each participant. The realignment parameters were used as covariates to account for motion-related noise signals. A high-pass filter with a cut-off period of 128 s was used to eliminate the artifactual low-frequency trend. Serial autocorrelation was accounted for using a first-order autoregressive model. For the second-level random-effects analysis, contrast images of the main effect of stimulus type (dynamic expression versus dynamic mosaic) were entered into a two-sample *t*-test using age as a covariate. The contrast of interest was YG versus controls. To ensure valid activity for dynamic facial expressions, the simple main effect of stimulus type was additionally tested in each group. If voxels reached the extent threshold of *P* < 0.05 with family-wise error (FWE) correction for the whole brain with a cluster-forming threshold of *P* < 0.001 (uncorrected), then they were deemed to be significant.

Brain structures were labeled anatomically and identified according to Brodmann’s areas using the Automated Anatomical Labeling Atlas (51) and Brodmann Maps (Brodmann.nii), respectively, with the MRIcron tool.^2^

#### 2.6.4. Structural MRI

All images were analyzed using the Computational Anatomy Toolbox CAT12^3^ of SPM12 with the default settings. All structural T1 images were segmented into gray matter, white matter, and cerebrospinal fluid by an adaptive maximum *a posteriori* (AMAP) approach (52). The homogeneity intensity of each image was modeled as a slowly varying spatial function and corrected in the AMAP estimate. These segmented images were used for partial volume estimation using a simple model with mixed tissue types to improve segmentation (53). A spatially adaptive non-local means denoising filter was applied to handle spatially varying noise levels (54). A Markov random field cleanup was employed to improve image quality. Gray matter images in native space were subsequently normalized to the standard stereotactic space defined by the MNI using diffeomorphic anatomical registration with the exponentiated Lie algebra algorithm approach (55). The resulting normalized gray matter images were modulated using Jacobian determinants with non-linear warping only to exclude the effect of total intracranial volume. Finally, the normalized modulated gray matter images were resampled to a resolution of 1.5 × 1.5 × 1.5 mm and smoothed using an 8-mm FWHM isotropic Gaussian kernel based on the recommendation for the VBM method, where FWHM is typically between 4 and 12 mm (56).

To identify the brain regions associated with gray matter volume difference between YG and controls, we performed a two-sample *t*-test with the covariates of total brain volume and age; the contrast of YG versus controls was tested. Voxels were deemed significant if they reached the extent threshold of *P* < 0.05 following FWE correction for multiple comparisons over the search volume, with a cluster-forming threshold of *P* < 0.001 (uncorrected). First, we corrected for FWE for the entire brain. Then, we conducted small-volume correction for the anatomically defined PMv (i.e., the IFG opercular and triangular parts) and STS region (i.e., superior and middle temporal gyri) in the right hemisphere based on our interest. Brain structures were identified using the same method as the above-described functional image analysis.

## 3. Results

### 3.1. Behavior

**FIGURE 2:**
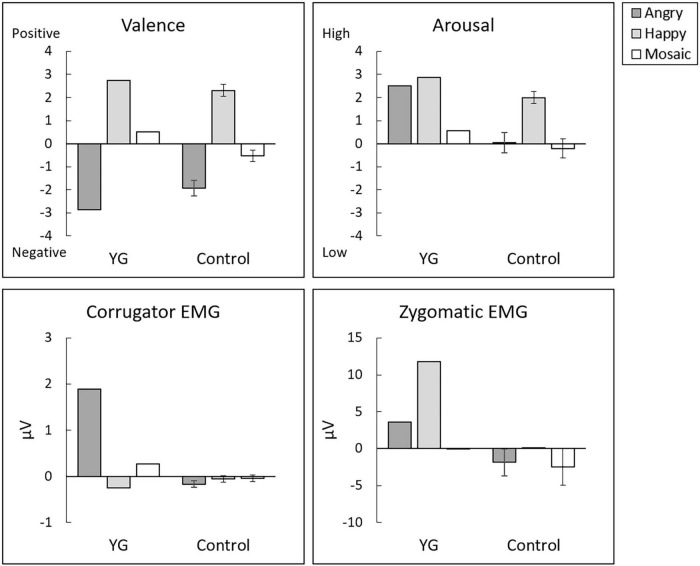
Mean (±*SE*) subjective ratings of experienced valence **(upper left)** and arousal **(upper right)** and electromyography (EMG) activity of the corrugator supercilii **(lower left)** and zygomatic major **(lower right)** muscles in response to dynamic angry expressions, dynamic happy expressions, and dynamic mosaics.

For the valence ratings (Figure 2, upper left), *t*-tests showed no significant difference between YG and controls (*t* = 0.71, *p* = 0.246, *z*_*cc*_ = 0.73). For the arousal ratings (Figure 2, upper right), *t*-tests revealed a significant difference between YG and controls (*t* = 2.12, *p* = 0.028, *z*_*cc*_ = 2.20), indicating higher arousal ratings for dynamic facial expressions in YG than in controls.

For corrugator supercilii EMG activity in response to angry expressions (Figure 2, lower left), *t*-tests revealed a significant group difference (*t* = 7.35, *p* < 0.001, *z*_*cc*_ = 7.63), indicating stronger facial mimicry responses to others’ angry expressions in YG than in controls. For zygomatic major EMG activity in response to happy expressions (Figure 2, lower right), *t*-tests revealed a significant group difference (*t* = 61.88, *p* < 0.001, *z*_*cc*_ = 64.22), indicating stronger facial mimicry responses to others’ happy expressions in YG than in controls. Visual inspection of the videos indicated that YG evidenced externally observable facial expressions congruent with the facial expressions of the stimuli. In the controls, no detectable facial reactions were systematically associated with the stimuli.

### 3.2. Regional brain activity

Contrasts between dynamic facial expression versus dynamic mosaic observations in YG and controls (Supplementary Figure 1 and Supplementary Tables 1, 2) revealed similar brain activity to those reported in previous studies [e.g., (23)]. Specifically, in both YG and controls, significant activity was detected in the right PMv and bilateral STS regions. However, these clusters in the PMv and STS region in the right hemisphere were more extended in YG than in controls.

**FIGURE 3:**
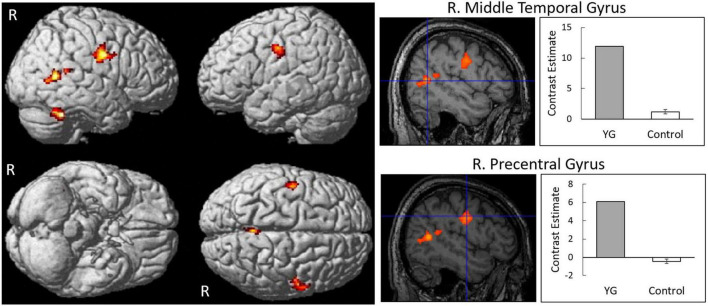
Statistical parametric maps indicating regions that were significantly more activated in YG than in controls in response to dynamic expressions versus dynamic mosaics. Areas of activation are rendered on spatially normalized brain **(left)** and spatially normalized magnetic resonance images of a representative participant **(middle)**. Blue crosses indicate activation foci in the group difference. Effect sizes **(right)** are indicated by mean (±*SE*) beta values of regions at the sites of activation foci. R, right.

The contrast between YG versus controls in terms of brain activity reacting to dynamic expressions versus dynamic mosaics revealed significantly stronger activity in some brain regions, including the PMv (covering the precentral gyrus and IFG) and STS region (covering the superior, middle, and inferior temporal gyri) in the right hemisphere (Figure 3 and Table 1). No other significant regions were detected in this contrast. There was no significantly stronger activity in controls than YG.

**TABLE 1:** Brain regions that were significantly more activated in YG than in controls in response to dynamic expressions versus dynamic mosaics.

Side	Region	BA	Coordinates			*T*-value	Cluster size
			*x*	*y*	*z*		(voxel)
**YG > Controls**							
R	Cerebellum	–	44	−50	−32	14.81	169
L	Supramarginal gyrus	43	−40	−16	28	7.95	189
R	Middle temporal gyrus	21	50	−54	8	7.81	244
R	Middle temporal gyrus	37	52	−62	6	5.77	
R	Precentral gyrus	4	48	−8	34	6.64	331
R	Precentral gyrus	6	58	2	30	6.19	
**Controls > YG**							
None							

BA, Brodmann’s area.

### 3.3. Regional gray matter volume

**FIGURE 4:**
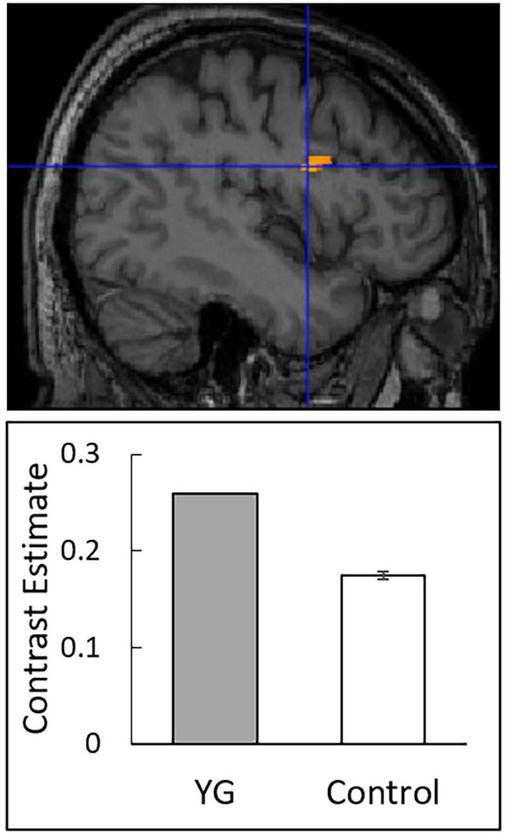
Statistical parametric maps indicating regions that showed significantly higher gray matter volume in YG than in controls. Significant areas are rendered on spatially normalized magnetic resonance images of a representative participant **(upper)**. A blue cross indicates the group difference focus. Effect sizes **(lower)** are indicated by mean (±*SE*) beta values at the focus.

The results revealed no significant difference in gray matter volume between YG and controls when FWE correction was applied for the entire brain. When we restricted our search volumes based on our interest in the MNS regions for facial expression processing (i.e., PMv and STS regions in the right hemisphere), there was a significant main effect for group in the right PMv (i.e., the IFG; peak: *x* = 44, *y* = 3, *z* = 23; *T* = 6.19; 96 voxels; Figure 4), with higher volume in YG than in controls.

## 4. Discussion

In this study, we investigated the behavioral and neural characteristics related to empathy in the Humanitude-care expert YG, and control participants. We acknowledge that testing only one Humanitude-care expert is a limitation of the study. Selection bias (57) was inevitable, and the generalizability of our findings thus remains to be tested (58). However, single-case research has often yielded valuable insights into our understanding of clinical and psychological phenomena and has complemented research using large groups (59–61). Further, to enhance the value and rigor of our work, we combined behavioral and neuroimaging methods (61, 62) and used validated statistical tools to compare the single case and controls (31). We believe that our results with an expert individual afford unique insights into Humanitude-care practitioners. Another limitation of this study is that we used a cross-sectional design in this study, and therefore cannot conclude causal relationships between behavioral or neural characteristics of YG and Humanitude care. It is possible that YG’s experience in elderly care using Humanitude techniques for more than 38 years has shaped his unique behavioral and neural characteristics. Alternatively, he may have an innate talent for empathic social interaction and selected an appropriate career to use his empathic abilities to help people with dementia. However, ample evidence indicates that becoming an expert generally requires practice rather than innate talent (63, 64), and some data suggest that this is also true among medical professionals (65, 66). Additionally, anecdotal records about YG’s life indicate that his first job was as a sports teacher, involving diving and swimming instruction. When YG started to care for people with dementia, he experienced many failures, and sought innovative care techniques. YG improved his care techniques gradually through interaction with more than 30,000 individuals with dementia. Based on this information, we speculate that our results at least partially reflect the experiences of Humanitude care for people with dementia. Future research comparing different stages of Humanitude-care expertise is warranted to test this idea.

Our behavioral results showed that YG exhibited stronger subjective arousal and facial mimicry than the controls. The control group did not show any clear pattern of facial mimicry, which is consistent with previous findings that facial mimicry may be decreased in response to the faces of other races than to those of the same race (67–69). Regardless, YG demonstrated clear facial mimicry. These results are consistent with findings that the experiences of Humanitude care enhance empathy in caregivers (10, 11) and that people with greater empathic traits report stronger shared emotional experiences (70–75) and show more evident facial mimicry (70–80). Together with these data, our results suggest that the experience of long-time Humanitude care improves behavioral (subjective and motor) characteristics associated with empathy.

Our functional MRI data showed that YG exhibited stronger MNS activity, specifically in the right PMv and STS region, in response to dynamic facial expressions than did controls. Our observed activation of the PMv and STS region in the right hemisphere is compatible with previous neuroimaging findings indicating that these regions were active during observation of dynamic facial expressions (21–24) and were the functional network used during processing of such expressions (22, 24). The heightened activities in the right PMv and STS and the facial mimicry of YG are also in agreement with previous findings that these regions were associated with facial EMG activity during observation of facial expressions (24, 81). Based on the assumption that YG has heightened empathy through Humanitude-care experience (10, 11), the results are consistent with previous neuroimaging findings that people with high empathic traits show stronger activity in the MNS during the observation of others’ facial or bodily actions (82–86). The data are also consistent with previous neuroimaging findings that expert groups of professional ballet dancers and professional pianists show stronger MNS activity during the observation of bodily actions related to their expertise (87–90). Other studies also found that non-expert participant groups who trained in dancing skills show enhanced MNS activity during the observation of dancing bodily actions (91, 92). Our data extend these findings and indicate that such an enhancement effect on MNS activity can be acquired through elderly care experiences, demonstrated through the observation of facial actions, and evaluated even in a single expert.

Our structural MRI data revealed that the gray matter volume of the right PMv was higher in YG than controls. The fact that YG evidenced enhancements in terms of both facial mimicry and the gray matter volume of the right PMv is in line with the finding of an earlier structural MRI study that gray matter volume in this region was increased in participants who identified emotions in facial expressions more accurately than others (93). As in the case of the functional MRI results, under the assumption that YG acquired heightened empathy through Humanitude-care experience (10, 11), the results are consistent with those of previous studies that people with greater empathic traits have increased gray matter volume in the PMv (94, 95). The results are also compatible with the previous findings that groups of musicians showed increased gray matter volume in the PMv, possibly reflecting their musical expertise (96–100), and that musical training increased gray matter volume in the PMv (99, 101, 102). Collectively, our results suggest that long-term experience of Humanitude care increases the gray matter volume of the MNS, to an extent detectable in a single participant.

Our data have a theoretical implication that Humanitude-care experience can improve behavioral (i.e., shared subjective emotional experience and facial mimicry) and neural (i.e., MNS activity and structure) characteristics associated with empathy. This result is notable, because Humanitude-care techniques do not explicitly offer instruction in these subjective, behavioral, and neural responses, although face-to-face interaction with eye contact at close distance is a pillar of Humanitude care (5). It may be difficult to implement natural facial mimicry deliberately, which is rapid (103) and automatic (104, 105). We speculate that, as Humanitude techniques are designed to create and maintain compassionate and respectful relationships between caregivers and people with dementia (2, 8), repeated Humanitude-care experience may develop empathic behavioral and neural characteristics in caregivers. In the general clinical care literature, several studies have found that empathic traits in caregivers generally produced good patient outcomes, such as patient satisfaction and improved cholesterol levels [e.g., (106); for reviews, see (107, 108)]. Some studies also showed that behaviors and experiences shared between caregivers and patients were associated with symptom reduction in those with chronic illnesses [e.g., (109); for a review, see (110)]. Interviews with numerous caregivers of people with dementia revealed that shared emotion was vital to maintaining relationships with people with dementia (111). Together with these data, our findings suggest that the empathic behavioral and neural characteristics of Humanitude caregivers may contribute to the positive effects of Humanitude care to reduce the behavioral and psychological symptoms of dementia [for a review, see (8)].

Our data also provide a unique perspective on the potential of Humanitude care. The results showed heightened facial mimicry and increased activity and structure in the MNS region in a Humanitude-care expert. Previous studies have reported that individuals with autism spectrum disorder (ASD) show weakened facial mimicry (112, 113) and reduced activity [e.g., (22, 114–116)] and gray matter volume [e.g., (117–120)] in the MNS region [for a review, see (121)]. Some studies showed that social skill training can improve social functioning in individuals with ASD [for a review, see (122)]; however, further research to develop effective training techniques is warranted. The results of this study suggest that performing Humanitude care (i.e., social interaction using Humanitude care techniques) may have the potential to enhance facial mimicry behaviors and MNS activity and structure in individuals with ASD. It would be interesting to test this hypothesis in a future study.

Apart from the intrinsic limitations of the study design discussed above, there were several other limitations. First, we presented only Japanese faces to Caucasian participants. We expected that such outgroup faces would reveal differences between YG and controls, as the effect of empathy was reportedly evident in positive attitudes to outgroup members (27, 28). However, whether Caucasian faces might produce different results and whether our current results can be generalized to faces of other races remain to be tested. Future studies should investigate the behavioral and neural responses to faces of other races by Humanitude-care experts. Second, our control sample size was small; we detected only a large effect size. Future studies with larger samples may reveal more behavioral and neural characteristics associated with Humanitude-care expertise. Finally, although we found heightened empathic behavioral and neural characteristics in a Humanitude-care expert, it remains unknown how these characteristics may contribute to the positive effects of Humanitude care, including reductions in the behavioral and psychological symptoms of dementia (8). Further work is warranted to investigate causal relationships between the empathic characteristics of Humanitude-care experts and the care effects.

In conclusion, our behavioral results demonstrated that the Humanitude expert YG experienced stronger shared emotion and exhibited more evident facial mimicry compared with controls. Our functional MRI data demonstrated that YG showed enhanced activity in the MNS (the PMv and STS region in the right hemisphere) in response to dynamic facial expressions, compared with controls. Structural MRI data revealed higher regional gray matter volume in the right PMv in YG than in controls. These results imply that Humanitude-care experts have behavioral and neural characteristics associated with empathic social interactions.

## Data availability statement

The data supporting the conclusions of this article will be made available by the authors, excluding any identifiable data.

## Ethics statement

The studies involving human participants were reviewed and approved by the Ethics Committee of the Unit for Advanced Studies of the Human Mind, Kyoto University, Japan. The patients/participants provided their written informed consent to participate in this study. Written informed consent was obtained from the individual(s) for the publication of any potentially identifiable images or data included in this article.

## Author contributions

WS, AN, SY, and MH designed the research, and obtained the data. WS and TK analyzed the data. All authors wrote the manuscript, read, and approved the final manuscript.

## Footnotes

1
http://www.fil.ion.ucl.ac.uk/spm

2
http://www.mccauslandcenter.sc.edu/mricro/mricron/

3
http://www.neuro.uni-jena.de/cat/
