# Supplementary material for: Behavioral and neural underpinnings of empathic characteristics in a Humanitude-care expert
Source: Front Med (Lausanne). 2023 May 25;10:1059203. doi: 10.3389/fmed.2023.1059203 (PMC10248535; doi:10.3389/fmed.2023.1059203)
Supplement: Supplementary file 1 [file Data_Sheet_1.docx]

Supplementary Material


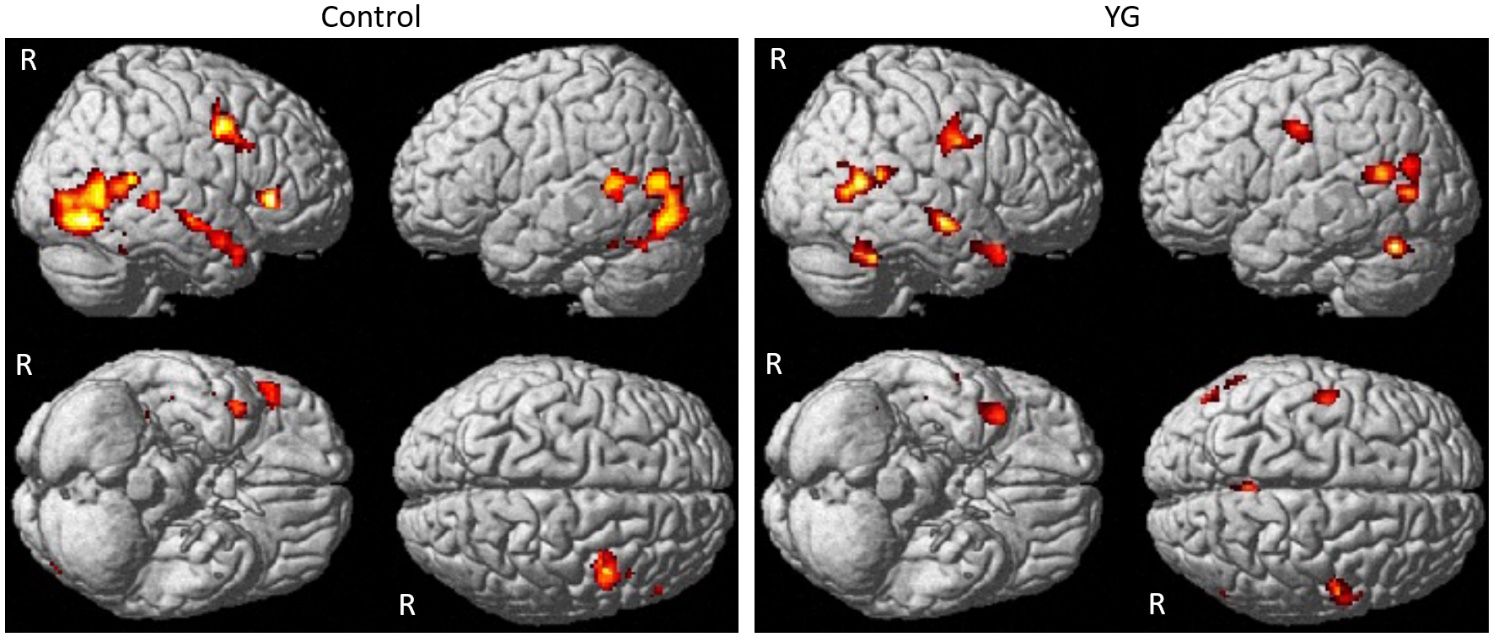


## Figure S1. Statistical parametric maps indicating regions that were significantly more activated in controls (left) and YG (right) in response to dynamic expressions versus dynamic mosaics. Areas of activation are rendered on the spatially normalized brain. R = right.

**Table S1.** Brain regions that were significantly more activated in response to dynamic expressions versus dynamic mosaics in controls.

| Side | Region | BA |  | Coordinates | | | *T*-value | Cluster size |
| --- | --- | --- | --- | --- | --- | --- | --- | --- |
|  |  |  |  | *x* | *y* | *z* |  | (voxel) |
| R | Middle temporal gyrus | 20 |  | 52 | -12 | -12 | 10.69 | 303 |
| R | Superior temporal gyrus | 38 |  | 42 | 10 | -26 | 5.75 |  |
| R | Precentral gyrus | 4 |  | 34 | 4 | 40 | 9.60 | 466 |
| R | Fusiform gyrus | 19 |  | 48 | -70 | -12 | 9.18 | 1365 |
| R | Inferior occipital gyrus | 19 |  | 42 | -78 | -10 | 8.88 |  |
| R | Middle temporal gyrus | 37 |  | 50 | -62 | 0 | 7.51 |  |
| R | Middle temporal gyrus | 21 |  | 58 | -52 | 6 | 7.45 |  |
| R | Fusiform gyrus | 37 |  | 38 | -60 | -10 | 6.07 |  |
| R | Inferior frontal gyrus | 47 |  | 48 | 30 | -2 | 9.15 | 159 |
| L | Middle temporal gyrus | 37 |  | -46 | -68 | 10 | 7.27 | 829 |
| L | Inferior occipital gyrus | 19 |  | -44 | -76 | -10 | 7.10 |  |
| L | Middle temporal gyrus | 21 |  | -48 | -48 | 12 | 7.15 | 207 |

BA = Brodmann’s area.

**Table S2.** Brain regions that were significantly more activated in response to dynamic expressions versus dynamic mosaics in YG.

| Side | Region | BA |  | Coordinates | | | *T*-value | Cluster size |
| --- | --- | --- | --- | --- | --- | --- | --- | --- |
|  |  |  |  | *x* | *y* | *z* |  | (voxel) |
| R | Middle temporal gyrus | 22 |  | 54 | -10 | -12 | 17.56 | 190 |
| R | Cerebellum | - |  | 44 | -50 | -32 | 15.19 | 218 |
| R | Middle temporal gyrus | 21 |  | 52 | -54 | 10 | 9.02 | 524 |
| R | Middle temporal gyrus | 37 |  | 52 | -62 | 4 | 7.78 |  |
| L | Middle temporal gyrus | 37 |  | -38 | -64 | 4 | 8.78 | 387 |
| L | Middle temporal gyrus | 39 |  | -48 | -72 | 18 | 7.61 |  |
| L | Supramarginal gyrus | 43 |  | -40 | -16 | 28 | 7.57 | 242 |
| R | Precentral gyrus | 4 |  | 48 | -8 | 34 | 6.44 | 292 |
| R | Temporal pole | 28 |  | 40 | 18 | -26 | 6.14 | 142 |

BA = Brodmann’s area.
